# The PANDA study: a randomized phase II study of first-line FOLFOX plus panitumumab versus 5FU plus panitumumab in *RAS* and *BRAF* wild-type elderly metastatic colorectal cancer patients

**DOI:** 10.1186/s12885-018-4001-x

**Published:** 2018-01-25

**Authors:** Francesca Battaglin, Marta Schirripa, Federica Buggin, Filippo Pietrantonio, Federica Morano, Giorgia Boscolo, Giuseppe Tonini, Eufemia Stefania Lutrino, Jessica Lucchetti, Lisa Salvatore, Alessandro Passardi, Chiara Cremolini, Ermenegildo Arnoldi, Mario Scartozzi, Nicoletta Pella, Luca Boni, Francesca Bergamo, Vittorina Zagonel, Fotios Loupakis, Sara Lonardi

**Affiliations:** 1 Istituto Oncologico Veneto - IRCCS, S.C. Oncologia Medica 1, Dipartimento di Oncologia Clinica e Sperimentale, Via Gattamelata, 64, 35128 Padova, Italy; 2 Fondazione IRCCS Istituto Nazionale Tumori, Dipartimento di Oncologia Medica, Via Venezian, 1, 20133 Milan, Italy; 3 Azienda Unita Locale Socio-Sanitaria N°3 Serenissima – Distretto Mirano, Dolo, Noale – Dipartimento di Scienze Mediche, U.O.C. Oncologia ed Ematologia Oncologica, Via Don G. Sartor, 4, 30035 Mirano, VE Italy; 4 Policlinico Universitario Campus Bio-Medico, U.O.C. Oncologia Medica, Via Alvaro del Portillo, 200, 00128 Rome, Italy; 5 Ospedale Senatore A. Perrino, U.O.C. Oncologia Medica, Strada Statale 7 (Appia), 72100 Brindisi, Italy; 6 U.O. Oncologia Medica, Dipartimento di Medicina, Ospedale Universitario Policlinico Tor Vergata, Università degli Studi di Roma Tor Vergata, Viale Oxford, 81, 00133 Rome, Italy; 7 Oncologia, Azienda Ospedaliera Universitaria Integrata di Verona, Piazzale Scuro, 10, 37134 Verona, Italy; 8 IRCCS Istituto Scientifico Romagnolo per lo Studio e la Cura dei Tumori (I.R.S.T.), U.O. Oncologia Medica, Via P. Maroncelli, 40, 47014 Meldola, FC Italy; 9 Azienda Ospedaliero Universitaria Pisana, U.O. Oncologia Medica 2 Universitaria, Via Roma, 67, 56126 Pisa, Italy; 10 ASST Papa Giovanni XXIII, U.O. Oncologia Medica, Piazza OMS, 1, 24127 Bergamo, Italy; 11 U.O. Oncologia Medica, Azienda Ospedaliero-Universitaria di Cagliari e Università di Cagliari, via Ospedale, 54, 09124 Cagliari, Italy; 12 Azienda Sanitaria Universitaria Integrata di Udine, Dipartimento di Oncologia, Piazzale Santa Maria della Misericordia, 15, 33100 Udine, Italy; 13 Centro Coordinamento Sperimentazioni Cliniche – Istituto Toscano Tumori, Via Taddeo Alderotti, 26/N, 50139 Florence, Italy

**Keywords:** Elderly, Metastatic colorectal cancer, RAS, BRAF, Panitumumab, G8, CRASH, Clinical trial

## Abstract

**Background:**

Few data are available regarding the treatment of metastatic colorectal cancer elderly patients with anti-EGFR agents in combination with chemotherapy. FOLFOX plus panitumumab is a standard first-line option for *RAS* wild-type metastatic colorectal cancer. Slight adjustments in chemo-dosage are commonly applied in clinical practice to elderly patients, but those modified schedules have never been prospectively tested. Clinical definition of elderly (≥70 years old) patients that may deserve a more or less intensive combination therapy is still debated. Several geriatric screening tools have been developed to predict survival and risk of toxicity from treatment. Among those, the G8 screening tool has been tested in cancer patients showing the strongest prognostic value for overall survival, while the CRASH score can stratify patients according to an estimated risk of treatment-related toxicities.

**Methods:**

The PANDA study is a prospective, open-label, multicenter, randomized phase II trial of first-line therapy with panitumumab in combination with dose-adjusted FOLFOX or with 5-fluorouracil monotherapy, in previously untreated elderly patients (≥70 years) with *RAS* and *BRAF* wild-type unresectable metastatic colorectal cancer. *RAS* and *BRAF* analyses are centralized. Geriatric assessment by means of G8 and CRASH score is planned at baseline and G8 will be re-evaluated at disease progression. The primary endpoint is duration of progression-free survival in both arms. Secondary endpoints include prospective evaluation of the prognostic role of G8 score and the correlation of CRASH risk categories with toxicity.

**Discussion:**

The PANDA study aims at exploring safety and efficacy of panitumumab in combination with FOLFOX or with 5FU/LV in elderly patients affected by *RAS* and *BRAF* wild-type metastatic colorectal cancer, to identify the most promising treatment strategy in this setting. Additionally, this is the first trial in which the prognostic role of the G8 score will be prospectively evaluated. Results of this study will drive further experimental developments for one or both combinations.

**Trial Registration:**

PANDA is registered at Clinicaltrials.gov: NCT02904031, July 11, 2016. PANDA is registered at EudraCT-No.: 2015–003888-10, September 3, 2015.

## Background

Aging population is highly represented among metastatic colorectal cancer (mCRC) patients [1]. However, the therapeutic decision making in patients older than 70 years of age is still a debated issue due to the paucity of trial-based recommendations.

The clinical definition of elderly (over 70 years) CRC patients that may deserve a more or less intensive combination therapy is still debated. A reasonable approach for defining candidates to different treatment intensity is, in this setting, to consider a cut-off of 75 years old combined with Eastern Cooperative Oncology Group – performance status (ECOG PS) assessment, in order to discriminate patients eligible to combination therapies from patients eligible to less intensive treatment.

In the daily clinical practice, treatment choices are mainly driven by data from retrospective studies, post-hoc or pooled analyses of randomized clinical trials or meta-analyses. However, those results do not necessarily reflect the general mCRC population and are often limited by potential confounding factors [2, 3]. Thus, an individualized treatment approach is usually adopted in this setting, after an in-depth evaluation of biological age, performance status, comorbidities, polypharmacy, social support, global functioning and cognitive abilities, and consequently the risk of experiencing adverse events and decreased quality of life [4].

Currently, in elderly patients, fluoropyrimide-based monotherapy plus bevacizumab, irrespectively of the molecular status of *RAS* (*Rat sarcoma viral oncogene homolog*), is a reasonable upfront treatment based on the result of the phase III open-label AVEX trial. Patients aged ≥70 years were randomized to receive capecitabine with or without bevacizumab. Among 280 randomized patients, the addition of bevacizumab improved progression free survival (PFS), the primary endpoint (9.1 versus 5.1 months; Hazard Ratio (HR) 0.53, 95% Confidence Interval (CI) 0.41–0.69; *p* < 0.0001), as well as the overall response rate (ORR) (19% versus 10%; *p* = 0.04); the difference in overall survival (OS), a secondary end point, was not statistically significant (20.7 months versus 16.8 months; HR 0.79, 95% CI 0.57–1.09; *p* = 0.18) [5].

Data regarding the adoption of a doublet chemotherapy regimen derive from the MRC FOCUS2 and the FFCD 2001–2002 studies [6, 7].

The MRC FOCUS2 was the first randomized clinical trial conducted specifically among elderly and frail untreated mCRC patients, considered unfit for full-dose chemotherapy, with the aim to investigate reduced-dose chemotherapy options. Patients (*n* = 459; 22% < 70 years; 35% 70–75 years, and 43% > 75 years) was randomized to receive treatment with 5-fluorouracil (5-FU)/leucovorin (LV), simplified FOLFOX (folinic-acid, 5-FU, oxaliplatin), capecitabine, or CAPOX/XELOX (capecitabine, oxaliplatin), at 80% of the standard drug doses. The addition of oxaliplatin versus no oxaliplatin resulted in a non-statistically significant trend toward improvement in PFS (median 5.8 versus 4.5 months; HR 0.84, 95% CI: 0.69–1.01, *p* = 0.07), an improvement in overall response rate (ORR: 13% versus 35%; *p* < 0.001) with a lack of benefit in OS. No significant differences in adverse events were observed in patients receiving the combination treatment compared to those receiving a monotherapy. Overall, the MRC FOCUS2 data showed that the addition of reduced-dose oxaliplatin to fluoropyrimidine-based therapy is feasible [6].

The adoption of an irinotecan-based first-line chemotherapy in mCRC patients ≥75 years was evaluated in the phase III FFCD 2001–2002 study. Among 282 randomized patients, those receiving irinotecan plus 5FU/LV showed a significant benefit, over those who received 5-FU/LV alone, in terms of ORR (46.3% versus 27.4%; OR 2.3, 95% CI: 1.4–3.8, *p* = 0.001), a non-significant benefit in terms of PFS (7.3 versus 5.2 months; HR 0.84, 95% CI: 0.66–1.07, *p* = 0.15), and no benefit in terms of OS. Of note, geriatric screening tools were adopted to perform prognostic factor analyses for treatment safety and baseline decrements in cognitive function and Instrumental Activities of Daily Living (IADLs) predicted for grade 3 to 4 toxicity and risk for hospitalization [7].

Epidermal growth factor receptor (EGFR) signalling plays a key role in CRC development and EGFR inhibitors (cetuximab and panitumumab) are well established therapeutic agents in mCRC treatment [8, 9]. From early evidence of a potential subgroup effect in anti-EGFRs activity to post-hoc analyses of randomized trials, *Kirsten rat sarcoma* (*KRAS*) exon 2 and subsequently *KRAS* exons 3 and 4 and *NRAS* exon 2, 3 and 4 mutations have been identified as negative predictive biomarkers for anti-EGFRs activity. Currently, every patient considered for an anti-EGFR therapy must undergo an extended *RAS* mutational testing, including *KRAS* and *NRAS* codons 12, 13 of exon 2; 59, 61 of exon 3; and 117 and 146 of exon 4. Anti-EGFRs treatment is restricted to all *RAS* wild-type patients [10]. However, despite being molecularly selected according to regulatory guidelines, several patients with a *RAS* wild-type mCRC do not benefit from anti-EGFR agents, implying that other mutations/mechanisms of resistance can have an impact on anti-EGFRs activity. *V-Raf murine sarcoma viral oncogene homolog B1* (*BRAF*) V600E mutation is one of these, and available data support the use of *BRAF* as a negative predictive biomarker in clinical practice [11–13].

Although FOLFOX plus panitumumab is a standard first-line therapy option for *RAS* wild-type untreated mCRC patients [14], data on the adoption of anti-EGFRs in elderly mCRC patients are scarce and mostly derived from retrospective or small prospective studies of molecularly unselected patients. Slight adjustments in chemo-dosage are commonly applied in routinely practice to elderly patients, but those modified schedules have never been prospectively tested.

In the subgroup analysis of *RAS* wild-type patients from PRIME study the addition of panitumumab to the first-line FOLFOX-4 showed a benefit over FOLFOX-4 in the subset of patients aged more than 65 years (*n* = 188), in terms of OS (26.6 versus 17.4 months; HR 0.78, 95% CI 0.58–1.09), PFS (9.7 versus 9.2 months; HR 0.88, 95% CI 0.65–1.19) and ORR (49% versus 42%) and did not raise any safety concerns. Although the numbers are small, these data support the fact that there is no evidence of a negative interaction between age and treatment efficacy. The analysis, however, was conducted with an age cut-off of 65 years while the analysis of efficacy in the > 75 years population was limited by patient numbers (*n* = 34) to draw conclusions [15], leaving the question of combined treatment plus panitumumab in properly-defined elderly patients still open.

Similarly, in a small phase II trial, enrolling elderly (age ≥ 70 years) patients considered not candidates for chemotherapy, in the *RAS* wild-type subgroup (*n* = 15), the first-line monotherapy with panitumumab seemed to be effective (overall response rate 13.3%, median PFS: 7.9 months; median OS: 12.3 months) and well-tolerated [16]. Encouraging data were also reported in another study investigating panitumumab monotherapy in molecularly selected *RAS* and *BRAF* wild-type frail elderly patients deemed unfit for chemotherapy or irinotecan-based doublets [17].

It is crucial, thus, to prospectively explore the efficacy of different chemotherapy backbones in combination with panitumumab as first-line treatment in this setting of mCRC patients. Moreover, *RAS* and *BRAF* testing should be definitively proven as clinically useful in frail/very elderly patients, as the addition of anti-EGFRs to chemotherapy could confer a survival advantage and a significant improvement of quality of life in this subgroup of patients.

Several geriatric assessment methods have been developed to help driving treatment choices in elderly patients and to detect disabilities and comorbidities that may potentially contribute to an older patient’s vulnerability predisposing poor outcome and treatment complications. Among them, the G8 screening tool has been tested in cancer patients showing the strongest prognostic value for overall survival [18–20]; the CRASH score is able to stratify patients according an estimated risk of treatment-related toxicities [21].

On the basis of these considerations, we designed the present randomized phase II trial of first-line therapy panitumumab in combination with simplified FOLFOX schedule or with 5-FU/LV alone, in previously untreated elderly patients with *RAS* and *BRAF* wild-type unresectable mCRC.

## Methods/Design

### Aim

The main objective of this trial is to study the efficacy of panitumumab in combination with FOLFOX and with 5-FU/LV in elderly patients with *RAS* and *BRAF* wild-type mCRC.

### Trial design

**Fig. 1:**
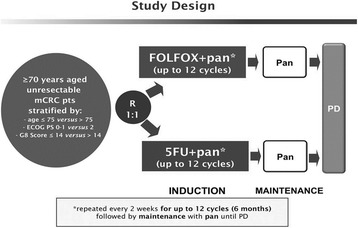
Study Design. *Pan = panitumumab

This is a prospective, open-label, multicenter phase II randomized trial in which initially unresectable and previously untreated *RAS* and *BRAF* wild-type mCRC elderly patients are randomized to receive FOLFOX plus panitumumab for up to 12 cycled followed by panitumumab maintenance until progressive disease (arm A), or 5FU/LV plus panitumumab for up to 12 cycled followed by panitumumab maintenance (arm B) until progressive disease (Fig. 1). A list of participating centers is provided in Table 1.

**Table 1:** Participating Centers

Principal Investigator	Site Name	City
Maura Rossi	Ospedale SS Antonio e C Arrigo	Alessandria
Angela Buonadonna	Centro di Riferimento Oncologico	Aviano
Nicola Silvestris	Istituto Tumori “Giovanni Paolo II”	Bari
Fable Zustovich	Ospedale San Martino ULSS 1 Dolomiti	Belluno
Ermenegildo Arnoldi	ASST Papa Giovanni XXIII	Bergamo
Giordano Beretta	Humanitas Gavazzeni	Bergamo
Claudio Graiff	Ospedale di Bolzano. Azienda Sanitaria dell’Alto Adige	Bolzano
Alberto Zaniboni	Fondazione Poliambulanza Istituto Ospedaliero	Brescia
Saverio Cinieri	Ospedale “Senatore A. Perrino”	Brindisi
Mario Scartozzi	Azienda Ospedaliero Universitaria di Cagliari Policlinico “Duilio Casula” Monserrato	Cagliari
Andrea Mambrini	A.S.L. 1 Massa Carrara	Carrara
Roberto Bordonaro	A.R.N.A.S. Garibaldi P.O. Nesima	Catania
Alberto Morabito	Ospedale Civile Pietro Cosma	Cittadella / Camposampiero
Gianluca Tomasello	Istituti Ospitalieri di Cremona	Cremona
Cristina Granetto	Azienda Sanitaria Ospedaliera Santa Croce e Carle	Cuneo
Carlo Milandri	A.U.S.L. 11 Empoli	Empoli
Francesca Vastola	Ospedale *M. Teresa* di Calcutta ULSS 17	Este/Monselice
Rosa Rita Silva	Ospedale E. Profili - Area vasta 2 ASUR	Fabriano
Rodolfo Mattioli	Presidio Ospedaliero Santa Croce	Fano
Davide Pastorelli	Ospedale di Feltre	Feltre
Antonio Frassoldati	Azienda Ospedaliero Universitaria Sant’Anna di Ferrara	Ferrara
Lorenzo Antonuzzo	Azienda Ospedaliero-Universitaria Careggi	Firenze
Angela Stefania Ribecco	P.O. S. Giovanni di Dio	Firenze
Teresa Gamucci	Polo Oncologico Provinciale Frosinone Azienda Sanitaria Locale	Frosinone
Alberto Ballestrero	IRCCS AOU San Martino-IST	Genova
Matteo Clavarezza	E.O. Ospedali Galliera	Genova
Carmelo Bengala	A.U.S.L. 9 Grosseto Ospedale Misericordia	Grosseto
Carlo Aschele	Ospedale Sant’Andrea ASL 5 Spezzino	La Spezia
Silvana Leo	Ospedale Vito Fazzi	Lecce
Antonio Ardizzoia	A.O. Provincia di Lecco	Lecco
Cecilia Barbara	Ospedali Riuniti di Livorno	Livorno
Editta Baldini	Ospedale San Luca	Lucca
Giovanni Luca Frassineti	I.R.C.C.S. Istituto Scientifico Romagnolo per lo Studio e la Cura dei Tumori (I.R.S.T.)	Meldola
Andrea Luciani	Azienda Ospedaliera San Paolo	Milano
Filippo De Braud	Istituto Nazionale dei Tumori	Milano
Andrea Sartore Bianchi	Ospedale Niguarda	Milano
Luca Gianni	Ospedale San Raffaele	Milano
Giorgia Boscolo	ULSS 13 Mirano	Mirano
Chiara Carlomagno	Azienda Ospedaliera Universitaria Federico II	Napoli
Stefania Gori	Ospedale Sacro Cuore – Don Calabria	Negrar
Sara Lonardi	Istituto Oncologico Veneto I.R.C.C.S.	Padova
Livio Blasi	Ospedale “CIVICO - DI CRISTINA - BENFRATELLI”	Palermo
Silvia Brugnatelli	Fondazione I.R.C.C.S. Policlinico San Matteo	Pavia
Alfredo Falcone	Azienda Ospedaliero Universitaria Pisana	Pisa
Giacomo Allegrini	Ospedale Felice Lotti Pontedera	Pontedera
Samantha Di Donato	Ospedale Santo Stefano	Prato
Filippo Giovanardi	Azienda USL di Reggio Emilia Ospedale di Guastalla	Reggio Emilia
Emiliano Tamburini	Ospedale Infermi	Rimini
Domenico Cristiano Corsi	Ospedale San Giovanni Calibita Fatebenefratelli Isola Tiberina	Roma
Mario Roselli	Policlinico Universitario ‘Tor Vergata’,	Roma
Enrico Cortesi	Policlinico Umberto I	Roma
Giuseppe Tonini	Policlinico Unversitario Campus Bio-Medico	Roma
Marco Benasso	A.S.L. 2 Savonese	Savona
Claudio Vergani	Azienda Ospedaliera Istituti Clinici di Perfezionamento	Sesto S. Giovanni
Francesco Di Clemente	Ospedali Riuniti della Valdichiana Senese. A.U.S.L. 7 Siena	Siena
Guido Francini	Azienda Ospedaliera Senese	Siena
Alessandro Bertolini	Azienda Ospedaliera della Valtellina e della Valchiavenna	Sondrio
Francesco Leone	Istituto di Candiolo I.R.C.C.S.	Torino
Michela Frisinghelli	Ospedale Civile Santa Chiara	Trento
Adolfo Favaretto	Azienda ULSS 9 Treviso Ospedale Ca′ Foncello	Treviso
Nicoletta Pella	A.O. Universitaria Santa Maria della Misericordia	Udine
Giampaolo Tortora	Azienda Ospedaliera Universitaria Integrata	Verona
Giuseppe Aprile	Ospedale San Bortolo	Vicenza
Enzo Maria Ruggeri	Ospedale Belcolle Viterbo	Viterbo

### Study endpoints

The primary endpoint is Progression Free Survival defined as the time from randomization to the first documentation of objective disease progression or death due to any cause. Documentation of disease progressive disease is defined as per Response Evaluation Criteria in Solid Tumors (RECIST) 1.1 criteria [22] based on investigator assessment.

The secondary endpoints include OS, ORR, early tumor shrinkage (ETS), R0 Resection Rate, overall toxicity rate (OTR), Toxicity Rate and geriatric assessment by G8 and by CRASH.

OS is defined as the time from randomization to the date of death due to any cause. The objective Response Rate will be evaluated according to RECIST 1.1 based on investigator reported measurements.

Early Tumor Shrinkage Rate is defined as the percentage of patients, relative to the total of the enrolled subjects achieving a ≥ 20% decrease in the sum of diameters of RECIST target lesions at week 8 compared to baseline.

Adverse events are evaluated according to the National Cancer Institute Common Terminology Criteria for Adverse Events (NCI CTCAE) version 4.0 [23], during the induction and the maintenance phases of treatment.

Of note, this is the first trial in which the prognostic role of the G8 score will be prospectively evaluated. The assessment by G8 score is defined as the score resulting from the G8 screening tool dichotomizing patients in two groups (score ≤ 14 and > 14). Moreover, the Geriatric Assessment by CRASH score, defined as the identification of four categories of different toxicity risk (low, medium-low, medium-high, high), will be prospectively correlated with toxicity.

### Clinical setting

Metastatic colorectal cancer patients aged ≥70 years are evaluable for the inclusion in the present study, an ECOG PS of 1 or 2 is required for patients aged 70 to 75 years, while an ECOG PS of 0 to 1 is required for patients aged more than 75 years.

Main eligibility criteria include:

- measurable disease according to RECIST version 1.1;
- centrally assessed wild-type *RAS* and *BRAF* status of primary colorectal cancer or related metastasis *(see following paragraph);*
- geriatric assessment by G8 screening tool and CRASH score *(see following paragraph)*;
- adequate bone marrow, liver, and renal function;
- no previous exposure to an oxaliplatin-containing adjuvant therapy. Previous adjuvant chemotherapy with fluoropyrimidines alone is allowed if more than 6 months have elapsed between the end of the adjuvant therapy and disease relapse.

Main exclusion criteria include:

- peripheral neuropathy of grade 1 or higher according to NCI CTCAE version 4.0;
- contraindications to study drugs.

Each patient’s eligibility is verified by using an electronic WEB-based system.

### Molecular assessment

Molecular testing of *RAS* and *BRAF* mutational status on tumor specimen for each patient is performed as a screening procedure and centralized at the Department of Surgical, Medical, Molecular Pathology and Critical Area, University of Pisa and Unit of Immunology and of Oncological Molecular Diagnostics, Department of Oncological Diagnostics, Oncology Institute of Veneto – IRCCS, Padova, Italy.

*KRAS* codon 12, 13, 59, 61, 117 and 146 mutations, *NRAS* codon 12, 13, 59, 61, 117 and 146 mutations and BRAF V600E mutation will be assessed by means of MALDI-TOF MassArray (Sequenom®).

### Geriatric assessment

Geriatric assessment by G8 screening tool is performed at baseline and at the time of disease progression. The G8 screening tool includes seven questions focusing on nutritional status, mobility, neuropsychological problems, medication use, self-rated health status and age. Each question provides from 2 to 4 possible answers and a score is assigned at any type of answer. The sum of the answers scores plus a score based on the age of the patient will be the final score. It ranges from 0 to 17 (Table 2).

**Table 2:** G8 Screening Tool

	Items	Possible Answers
A	Has food intake declined over the past 3 months due to loss of appetite, digestive problems, chewing or swallowing difficulties?	0: severe reduction in food intake 1: moderate reduction in food intake 2: normal food intake
B	Weight loss during the last 3 months?	0: weight loss > 3 kg 1: does not know 2: weight loss between 1 and 3 kg 3: no weight loss
C	Mobility	0: bed or chair bound 1: able to get out of bed/chair but does not go out 2: goes out
D	Neuropsychological problems	0: severe dementia or depression 1: mild dementia or depression 2: no psychological problems
F	Body Mass Index (weight in kg/height in m2)	0: BMI less than 19 1: BMI 19 to less than 21 2: BMI 21 to less than 23 3: BMI 23 or greater
G	Takes more than 3 medications per day	0: yes 1: no
H	In comparison with other people of the same age, how does the patient consider his/her health status?	0: not as good 0,5: does not know 1: as good 2: better
I	Age	0: > 85 1: 80–85 2: < 80

The CRASH screening tool is performed only at baseline by local investigators according to the online CRASH Score Calculator developed by Moffit Cancer Center [24]. This score stratifies patients in four risk categories of severe hematologic, non-hematologic and combined toxicities.

The hematologic sub-score is calculated by the sum of scores (ranged from 0 to 2) assigned to the following items: diastolic blood pressure, IADL, LDH, and MAX2 index.

The non-hematologic score is calculated by the sum of scores (ranged from 0 to 2) assigned to the following items: ECOG PS, Mini Mental State Exam (MMSE), Mini Nutritional Assessment and MAX2 index.

The combined score is calculated by the sum of scores (ranged from 0 to 2) assigned to the following items: diastolic blood pressure, IADL, LDH, ECOG PS, MMSE, MNA and MAX2 index (Table 3).

**Table 3:** CRASH Scoring System

Item	Score
Diastolic Blood Pressure	0: ≤72 mmHg 1: > 72 mmHg
IADL	0 = 8 1: < 8
LDH	0: ≤ 0.74 x ULN 2: > 0.74 x ULN
ECOG PS	0: 0 1: 1–2 2: 3–4
MMS	0: 30; 2: < 30
MNA	0: 28–30 2: < 28
Chemotherapy risk (MAX2)	0: arm B (FU/LV + Pani) 1: arm A (FOLFOX+Pani)

### Interventions

Patients considered eligible and who have signed a written informed consent are randomly assigned to one of the two treatment arms in a 1:1 ratio. Eligible patients will be stratified according to age (≤75 versus > 75 years), ECOG PS (0–1 versus 2) and G8 Score (≤14 versus > 14).

#### Arm A: FOLFOX plus panitumumab

Patients randomized to this arm receive FOLFOX-panitumumab every 2 weeks up to 12 cycles with the following schedule:

- Panitumumab 6 mg/kg iv over 60 min, day 1; if the first infusion is tolerated, then subsequent infusions may be administered over 30 to 60 min;
- Oxaliplatin 85 mg/sqm iv over 2 h, day 1;
- L-Leucovorin 200 mg/sqm iv over 2 h, day 1;
- 5-fluoruracil 2400 mg/sqm 48 h-continuous infusion, starting on day 1;

If no progression occurs, patients receive maintenance panitumumab at the same dose used at the last cycle of the induction treatment. Panitumumab is repeated biweekly until disease progression, unacceptable toxicity or patient’s refusal.

#### Arm B: 5-FU/LV plus panitumumab

Patients randomized to this arm receive 5FU-panitumumab every 2 weeks up to 12 cycles with the following schedule:

- Panitumumab 6 mg/kg iv over 60 min, day 1; if the first infusion is tolerated, then subsequent infusions may be administered over 30 to 60 min;
- L-Leucovorin 200 mg/sqm iv over 2 h, day 1;
- 5-fluoruracil 2400 mg/sqm 48 h-continuous infusion, starting on day 1;

If no progression occurs, patients receive maintenance panitumumab at the same dose used at the last cycle of the induction treatment. Panitumumab is repeated biweekly until disease progression, unacceptable toxicity or patient’s refusal.

Disease assessment is performed every 8 weeks by means of CT scan, according to RECIST 1.1 criteria.

Surgical radical resection of residual metastases in responsive patients is recommended and its feasibility should be evaluated every 2 months. After resection, patients will receive post-operative therapy up to 12 cycles of the same chemotherapy regimen plus panitumumab received before resection.

The second- and subsequent lines of treatment will be based on investigators’ choice.

### Statistical design

Assuming exponentially distributed event times, uniform accrual over time, no loss to follow-up and an expected median PFS time equal to or greater than 9.65 months with both experimental regimens, corresponding to a 6-month PFS probability ≥65%, a sample size of 90 patients in each arm will guarantee to the study a power equal to 90% for a one-sided Brookmeyer-Crowley test, with a type I error rate equal to 5%, against the null of a median PFS time equal to or less than 6 months.

The Kaplan-Meier approach will be used to estimate median PFS for each treatment arm. The hypothesis test will be conducted according to Brookmeyer-Crowley, comparing the cumulative hazard estimate at 6 months to the –log(0.50). No formal comparison between the results of the two treatment arms will be allowed.

All analyses of secondary endpoints will be descriptive only and no formal statistical comparisons will be made between the arms. Survival curves will be calculated according to Kaplan–Meier methods. Log-rank tests stratified by the same factors as used for randomization will also be performed, as well as multivariable models including all the significant baseline variables. The median event times and corresponding 2-sided 95% CI for the median will be provided.

The prognostic value of baseline G8 assessment for predicting patients’ outcome will be investigated through Cox proportional hazards model. The relationship between baseline data and patients’ survival will be graphically investigated. The prognostic value of changes of G8 score from baseline to disease progression will be similarly investigated. The analyses will be adjusted for known clinical prognostic factors. All analyses will be adjusted for multiple testing.

The association of G8 score with clinical outcome will be assessed in terms of the difference in OS in the two groups of patients (score ≤ 14 versus > 14) by means of log-rank test.

The association of CRASH score with toxicity will be assessed comparing G3–4 adverse events in the four risk categories (low, medium-low, medium-high and high risk) by means of chi-square test.

### Safety

All adverse events are recorded under the responsibility of the investigator in the subjects’ medical records and in electronic Case Report Forms (eCRFs), severity and relationship with the study treatment is assessed. Any medical condition that at the judgment of the Investigator may affect patients’ safety is a possible reason for treatment discontinuation.

An adverse event with the following characteristics: fatal, life threatening, requiring in-patient hospitalization or prolongation of existing hospitalization; resulting in persistent or significant disability/incapacity; congenital anomaly/birth defect or other significant medical hazard is defined as serious adverse event (SAE) and must be reported to the coordinating center within 24 h from its occurrence. The investigator should notify the Sponsor of all SAEs in accordance with local procedures, statutes and the European Clinical Trial Directive (where applicable). The Sponsor is responsible for the medical review of all SAEs and for their notification to the appropriate Ethics Committees, Competent Authorities and participating Investigators, in accordance with local requirements and the European Clinical Trial Directive.

### Data monitoring and quality assurance

Each participating Investigator is responsible for ensuring data quality as planned in the Data Validation Plan document. The coordinating Data Center is responsible of monitoring visits at the participating centers in order to verify consistency, completeness and accuracy of data and periodically issues Data Query Forms in case of inconsistent data. Investigators guarantee that all persons involved in this study respect the confidentiality of any information concerning the trial subject.

### Study schedule

The trial has started on July 2016. Study length is planned to be about 36 months, with an enrollment period of about 24 months and a minimum period of follow-up of 12 months. The estimated study completion date is July 2019 (final data collection date for primary outcome measure). Survival status will be collected until patients’ death.

### Coordination

Istituto Oncologico Veneto IOV-IRCCS is responsible for the overall coordination and management of the study on behalf of Gruppo Oncologico Nord-Ovest (G.O.N.O.) Cooperative Group.

### Ethics and regulatory considerations

This study is conducted in accordance with globally accepted standards of Good Clinical Practice and the latest version of the Declaration of Helsinki, and in agreement with local law(s) and regulation(s).

The study (Protocol version 2.2, April 18th 2016) was approved for all participating centers by AIFA, the Italian health authority (Agenzia Italiana del Farmaco) on May 23rd 2016 and registered on September 3rd 2015 at EudraCT database (EudraCT 2015–003888-10) and on July 11th 2016 at Clinicaltrials.gov (NCT02904031). Documented approval from appropriate IEC(s)/IRB(s) have been obtained for all participating centers before the start of the study.

Gruppo Oncologico Nord-Ovest (G.O.N.O.) Cooperative Group signed an insurance policy with the Company QBE Insurance to provide patients with compensation for any injury associated with administration of the study drugs and other aspects of the conduct of the trial.

In case of important protocol modifications (i.e. changes to eligibility criteria, outcomes, analysis) all necessary extensions, amendments and/or renewal will be notified to the IEC/IRB for approval and will be forwarded to the Sponsor.

All candidate patients provide their informed consent to study procedures before enrollment in the study. Investigators are responsible for informing each patient (or legally authorized representative) of the nature of the study, its purpose, the procedures involved, the expected duration, the potential risks and benefits involved and any discomfort it may entail.

## Discussion

Based on available data, fluoropyrimidine-based monotherapy plus bevacizumab is currently considered a standard upfront treatment for elderly mCRC patients, irrespectively of *RAS* status. To date, data on treatment of elderly patients with chemotherapy plus anti-EGFRs are scarce and rely on retrospective or non-randomized studies. While FOLFOX plus panitumumab is a standard first-line option for *RAS* wild-type mCRC, evidences about the combination of an anti-EGFR with a fluoropyrimidine-based monotherapy are lacking. The PANDA study aims at exploring the safety and efficacy of panitumumab in combination with FOLFOX or with 5-FU/LV in selected elderly patients with centrally-confirmed *RAS* and *BRAF* wild-type mCRC. The choice of a maintenance strategy with panitumumab monotherapy after an induction with chemotherapy plus panitumumab is based on recent literature evidences suggesting that maintenance therapy with single-agent anti-EGFR following chemotherapy plus anti-EGFR is not inferior to continuing treatment with chemotherapy plus anti-EGFR [25]. This strategy seems to be a reasonable option in a molecularly selected population of elderly patients in order to improve treatment tolerance and toxicity profile. The results of the present trial will help in driving further developments of one of the two combinations under study. Moreover, taking into account the complexity of clinical evaluation and treatment choices in elderly patients, geriatric assessment by means of G8 and CRASH score, two well renowned geriatric screening tools, has been integrated in the protocol procedures in order to ensure a comprehensive evaluation of patients before treatment start. The study will prospectively evaluate the association of these scores with clinical outcome and treatment-related severe toxicity, thus hopefully providing additional evidences for the use of these tools in everyday clinical practice to optimize cancer treatment of elderly patients.

Additionally, we expect that enrolling patients in the trial and performing *RAS/BRA*F testing in the study population will additionally give a more extensive view of the mutational incidence in this selected subset of patients in a real-life setting, improving our knowledge of the disease. At the same time, the collection of archival tissue alongside biological samples (blood and urine), which is planned as part of the study procedures, will contribute to create a valuable source for future analyses and research in this specific population.

Recently, primary tumor sidedness has emerged as a novel potential surrogate predictive marker for mCRCs treated with anti-EGFRs. Retrospective data from subgroup analyses of large randomized phase III trials presented in 2016 at the ASCO Annual Meeting and at the ESMO Congress, in fact, supported the evidence of a lack of benefit from anti-EGFR treatment in right-sided tumors. Conversely, patients with left-sided tumors showed better survival outcomes and treatment benefit from anti-EGFR therapies. These data have subsequently been confirmed by two large meta-analyses showing a significant benefit from first-line anti-EGFR treatment in RAS wild-type left-sided tumors opposite to right-sided ones [26, 27]. Despite the limitations of these unplanned retrospective subgroup analyses, data are consistent across several randomized trials, and support the rationale of avoiding anti-EGFRs in the first-line treatment for right-sided mCRCs when other options are available. Nevertheless, this evidence has not been prospectively validated and at the time of the design of our trial this topic was not under debate yet, thus primary tumor sidedness is not considered among inclusion/exclusion criteria. Data on primary tumor location are being collected in the study population and future analyses will help to further address this issue.

Additional observational studies are planned by our Institution to collect data on real life application of geriatric screening tools and efficacy/safety profile of combined treatment in elderly patients, and will integrate the current effort in defining the optimal treatment strategy for these patients [28].

In conclusion, results of our trial combined with those of other studies exploring different treatment combinations in a similar population (i.e. XELOX plus bevacizumab) [29] will contribute to create a body of evidences to better understand clinical and molecular features of mCRC in the elderly and guide clinical and therapeutic decisions in this complex setting.

## Abbreviations

5-FU: 5-fluorouracil
AIFA: Agenzia Italiana del Farmaco
BRAF: V-Raf murine sarcoma viral oncogene homolog B1
CAPOX/XELOX: Capecitabine, Oxaliplatin
CI: Confidence Interval
ECOG PS: Eastern Cooperative Oncology Group – performance status
eCRFs: electronic Case Report Forms
EGFR: Epidermal Growth Factor Receptor
ETS: Early tumor shrinkage
FOLFOX: Folinic-acid, 5-Fluorouracil, Oxaliplatin
G.O.N.O.: Gruppo Oncologico Nord-Ovest
HR: Hazard Ratio
IADLs: Instrumental Activities of Daily Living
LV: leucovorin
mCRC: metastatic Colorectal Cancer
MMSE: Mini Mental State Exam
NCI CTCAE: National Cancer Institute Common Terminology Criteria for Adverse Events
ORR: Overall Response Rate
OS: Overall Survival
OTR: Overall Toxicity Rate
PFS: Progression Free Survival
RAS: Rat sarcoma viral oncogene homolog
RECIST: Response Evaluation Criteria in Solid Tumors
SAE: Serious Adverse Event
